# Supplementary material for: Model-based lesion mapping of cognitive control using the Wisconsin Card Sorting Test
Source: Nat Commun. 2019 Jan 3;10:20. doi: 10.1038/s41467-018-07912-5 (PMC6318292; doi:10.1038/s41467-018-07912-5)
Supplement: Supplementary file 1 — Supplementary Information [file 41467_2018_7912_MOESM1_ESM.pdf]

Model-based lesion mapping of cognitive control using the Wisconsin Card Sorting Test

Gläscher, Adolphs, and Tranel

— Supplementary Information —

Supplementary Note 1: Scoring of the Wisconsin Card Sorting Test (WCST)

The WCST is commonly scored according to several test indices that can be derived from the score sheets [1]. Based on their ubiquitous usage in the field and the correlations among these test scores (see Figure 2 in the main text) we selected the following WCST scores for validating and comparing the results of the computational modeling:

1. Perseverative Errors (PSV): When, after a switch in sorting dimension, the participant continues to sort according to the previously correct dimension, this is scored as a perseverative error.
2. Non-perseverative Errors (NPSV): All errors that are not scored as perseverative.
3. Number of Categories Completed (NCAT): The number of the sorting dimensions that the participant completed successfully, defined by 10 consecutive correct responses, within the 128-card maximum.
4. Failure to maintain set (FSET): When a participant has completed 5 or more consecutively correct trials and then commits an error by switching to a different dimension (despite “right” feedback), this is scored as a failure to maintain set.
5. Trials to complete first Set (TRSET1): The total number of trials a participant used to complete the first sorting dimension (again, defined by 10 consecutive correct responses).

Supplementary Table 1. Demographic Information. Data are presented for each of the clusters identified by our 2-step cluster analysis (see Supplementary Note 7 and Supplementary Figures 9 and 10 and Supplementary Tables 3 and 4).

	Cluster				
	1	2c	2b	2c	3
**Gender (M/F)**	16/20	56/39	40/53	39/39	20/6
**Handedness (# Right/Mixed/Left)**	31/1/4	86/2/7	85/3/5	73/0/5	24/0/2
**Education (years)**	14.7 *	14.0	13.4	13.6	13.0
**Age at Testing**	41.4 *	47.1 *	54.4	50.4	53.0
**Etiology (N's)**					
Stroke	13	48	50	45	15
Resection	23	37	28	27	10
Encephalitis	0	3	1	1	1
Focal Contusion	0	2	6	2	0
Subarachnoid Hemorrhage	0	5	8	3	0

* indicates significant difference from other clusters

Supplementary Table 2. Other neuropsychological assessment data. Data are presented for each of the clusters identified by our 2-step cluster analysis (see Supplementary Note 7 and Supplementary Figures 9 and 10 and Supplementary Tables 3 and 4).

	Clusters				
	1	2c	2b	2c	3
**WAIS**					
Full-Scale IQ	106.97	104.94	99.75	100.48	95.47
Verbal IQ	105.28	103.89	99.30	100.06	97.52
Performance IQ	106.38	104.47	99.41	100.29	92.45
Verbal Comprehension	106.77	106.13	100.73	101.82	99.67
Perceptual Organization	109.72	107.11	103.36	103.94	96.46
Working Memory	99.23	101.15	97.12	97.98	91.42
Processing Speed	100.73	102.75	96.33	95.48	92.83
**WMS**					
Global Memory Index	100.16	102.58	102.33	95.84	92.00
Working Memory Index	102.61	105.25	99.45	101.97	89.88
**TMT** (seconds)					
Part A	26.48	32.73	40.53	39.23	50.05
Part B	58.97	77.07	96.70	95.40	129.29
B-A	32.48	44.34	56.16	56.17	79.24
**COWAT** (# words)	41.59	37.99	39.22	34.86	31.88
**CFT** (# correct/36)					
Copy	32.56	32.19	30.52	31.99	30.46
Recall	19.72	18.88	16.80	17.66	16.88
**WRAT Reading** (Std. Score)	102.16	98.28	98.31	95.22	88.42
**BNT** (# correct/60)	54.59	52.76	52.76	51.13	51.90

WAIS = Wechsler Adult Intelligence Scale-III, WMS = Wechsler Memory Scale-III, TMT = Trail-Making Test, COWAT = Controlled Oral Word Association Test, CFT = Complex Figure Test, WRAT = Wide Range Achievement Test-3, BNT = Boston Naming Test

Supplementary Note 2: Lesion Mapping

All neuroanatomical data were mapped using MAP-3[2]. Briefly, the visible lesion in each patient's MRI or CT scan was manually traced, slice by slice, onto corresponding regions of a single, normal reference brain (template brain) that has been used in prior studies with this method. Lesions were traced by an expert (Hanna Damasio) who has demonstrated high reliability [3]. This manual tracing was only done when confidence could be achieved for matching corresponding slices between the lesion brain and the reference brain. Thus, lesions were only mapped, if (i) they were clearly distinguishable from the (possibly dilated) ventricular system, (ii) there were no coexisting signs of cortical atrophy, and (iii) the MRI or CT scan showed no imaging artifact. Because the neuroanatomical data were manually traced to a stereotaxic template, no automated spatial normalization was required. The lesion maps for each patient were resampled to an isotropic voxel size of 1 mm^3, spatially smoothed with a 4-mm full-width-at-half-maximum (FWHM) Gaussian kernel, and binarized at a threshold of 0.2. Figure S1 presents the overall lesion density map for our 328 patients. Given that previous research on the WCST has often focused on the prefrontal cortex, the WCST is frequently administered to patients with lesions in the PFC. Not surprisingly, our lesion density map is biased toward the prefrontal cortex, and within it, more to the right than to the left hemisphere. The other brain regions with a dense lesion coverage are the anterior temporal lobes (ATL in both hemispheres). Lesion density influences statistical power to detect effects; we only analyzed voxels that had overlaps from at least 12 patients (see Methods). In the relevant Figures, regions with overlaps<12 are shaded in darker gray.

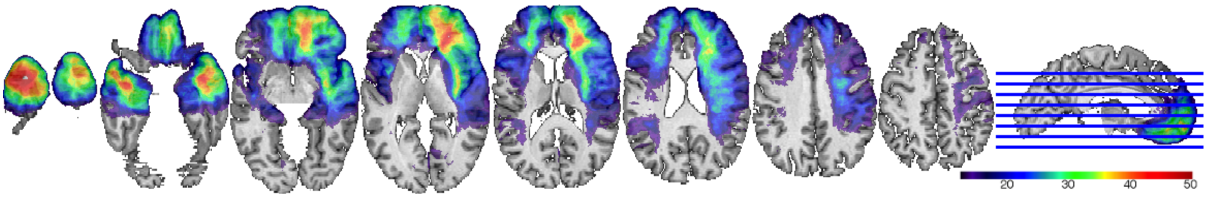

Supplementary Figure 1*.* *Lesion density map of 328 neurological patients who completed the WCST. The color scale encodes the number of patients in our sample with a lesion at a given voxel. Slices shown on the sagittal cut to the right correspond to the level of sections shown to the left.*

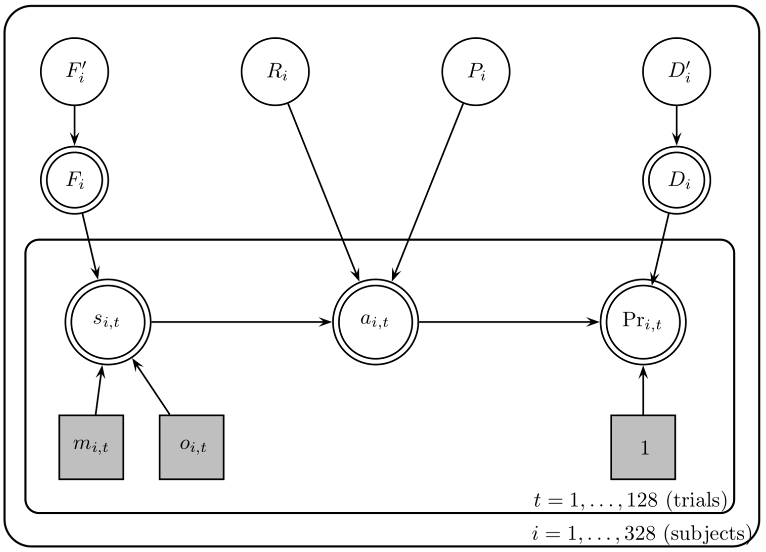

$$\{R, P, D', F'\} \sim \mathrm{Beta}(1,1)$$
$$\alpha_x = \mu_x \times \kappa_x$$
$$\beta_x = (1 - \mu_x) \times \kappa_x$$

$$\{D, F\} = \{D', F'\} \times 5$$

$$s_{i,t} = \begin{cases} m_{i,t} \times a_{i,t}^{F_i} & o_{i,t} = 1 \\ (1 - m_{i,t}) \times a_{i,t}^{F_i} & o_{i,t} = 0 \end{cases}$$

$$a_{i,t+1} = \begin{cases} a_{i,t} + R_i \times s_{i,t} & o_{i,t} = 1 \\ a_{i,t} + P_i \times s_{i,t} & o_{i,t} = 0 \end{cases}$$

$$Pr_{i,t} = \frac{m'_{i,t} a_{i,t}^{D_i}}{\sum a_{i,t}^{D_i}}$$

$$1 \sim Pr_{i,t}$$

Supplementary Figure 2. Graphical representation of the Bayesian model used to fit the Bishara model to the data. The model was estimated using Bayesian estimation[5] in a Markov Chain Monte Carlo sampling scheme using JAGS[6]. The notation in this figure follows the convention established by Lee and Wagenmakers[7]: observed nodes are shaded, continuous nodes are represented by circles, fully determined nodes are represented by double boundaries, and plates represent loops. All four free parameters are modeled as Beta distributions. While the parameters R and P have natural boundaries between 0 and 1, the parameters D and F are rescaled to a range between 0 and 5. These individual parameters are then used in the trial-by-trial model equations (see Methods).

Supplementary Note 3: Parameter Recovery Study

We created different combinations of parameter values: R (0.1,0.6,0.9), P (0.1,0.5,0.9), D (0.25,1.0), F (0.5,1.0). Systematic combinations of these values yielded 36 different parameter profiles. We simulated 60 virtual participants for each of these parameter combinations and fitted each of these simulated data sets individually with the Bishara et al. model[4] (shown in Figure S2) using Bayesian estimation[5].

In general, parameter recovery was very accurate as revealed by the mean squared error (MSE) between the mean of the maximum a posterior of the parameter distribution and the true parameter values used to generate the simulations. The model (estimates shown as bars) tends to underestimate the true parameter value (shown as a black line) for reward sensitivity (R) and punishment sensitivity (P), when the true parameter value is medium (0.5 or 0.6, R and P) or high (0.9, only P) when and the decision consistency parameter (D) is low (0.25).

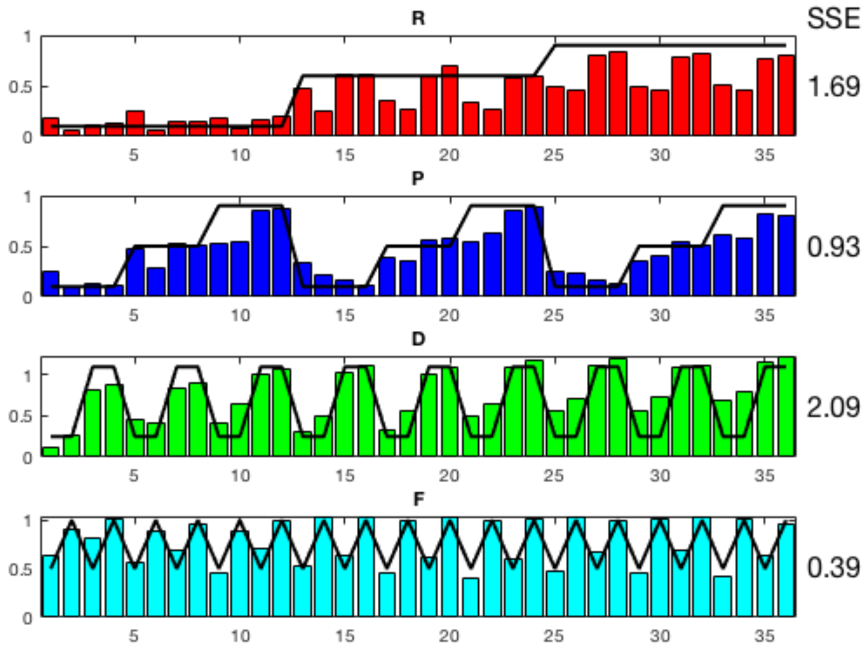

Supplementary Figure 3. Parameter recovery study. 36 parameter combinations for R, P, D, and F (shown as black lines) were used to simulate 60 virtual participants for each combination. We used the Bishara et al. model [4] to fit these virtual participants using Bayesian estimation. We extracted the maximum a posterior estimate of the individual parameter distribution and averaged them. These averages are shown as bar graphs in the 4 panels.

Supplementary Note 4: Simulation Study

To systematically assess the effects of different configurations of model parameters on perseverative errors, we conducted a grid search study varying all 4 parameters across a reasonable range (R and P from 0.1 to 0.9 in steps of 0.1, F and D from 0.5 to 4.5 in steps of 0.5). For each of the 6500 parameter combinations we simulated 100 data sets, calculated the WCST scores and averaged them across all 100 data sets.

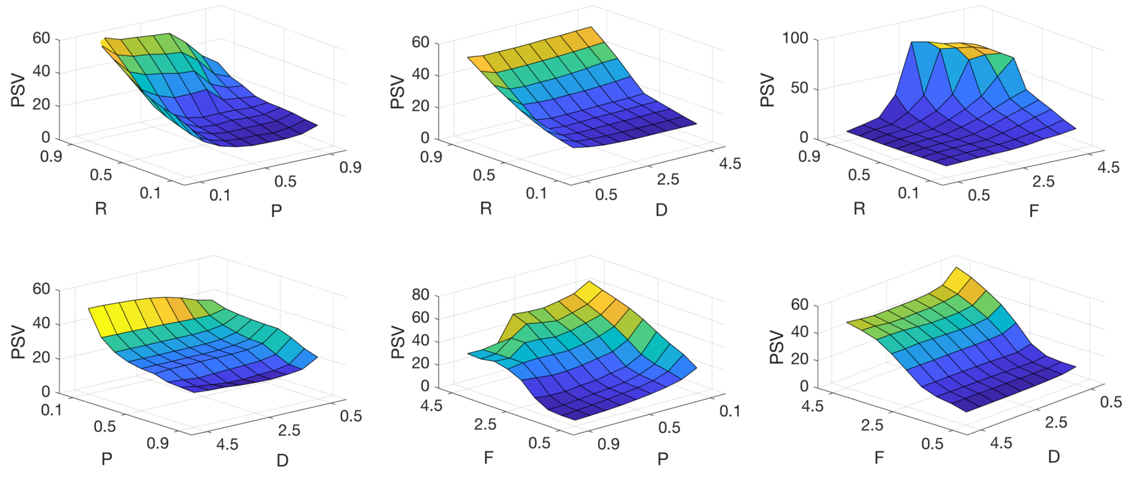

Supplementary Figure 4. Effects of variation in model parameters on perseverative errors. Each plot shows the average number of perseverative errors (PSV) as a function of the combination of 2 model parameters. The lowest point of this surface indicates the best parameter combination, which minimizes perseverative errors. This is the case when punishment sensitivity (P) is high, reward sensitivity (R) is low, decision consistency (D) is high, and attentional focusing (F) is low.

Supplementary Note 5: Voxel-based lesion symptom mapping

We used voxel-based lesion symptom mapping (VLSM) [8] to identify the anatomical lesion correlates associated with lower values of our 4 model parameters. We used the Brunner-Munzel (BM) test [9] at a threshold of 1% false discovery rate (FDR) [10], which corresponds to a critical Z-threshold of 3.1. This test is implemented in the "Nonparametric Mapping (NPM)" tool (version 16 May 2016) that is a part of the MRIcron software package [11] (http://www.mccauslandcenter.sc.edu/mricro/mricron/). We placed an initial lower bound on statistical power by including in all subsequent analyses only those voxels having a lesion overlap from at least 12 patients. Medina et al. [12] have shown that the Brunner-Munzel test (as implemented in an earlier version of NPM) results in inflated z-scores when less than 10 patients are included in the sample. This inflation at small sample sizes was corrected in later versions of the software that we used for these analyses.

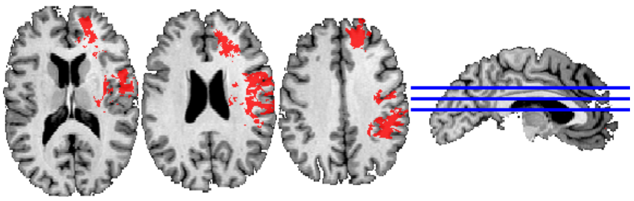

Supplementary Figure 5. VLSM analysis of the WCST score "Trial to complete 1st set (TRSET1), thresholded at 1% false discovery rate (FDR).

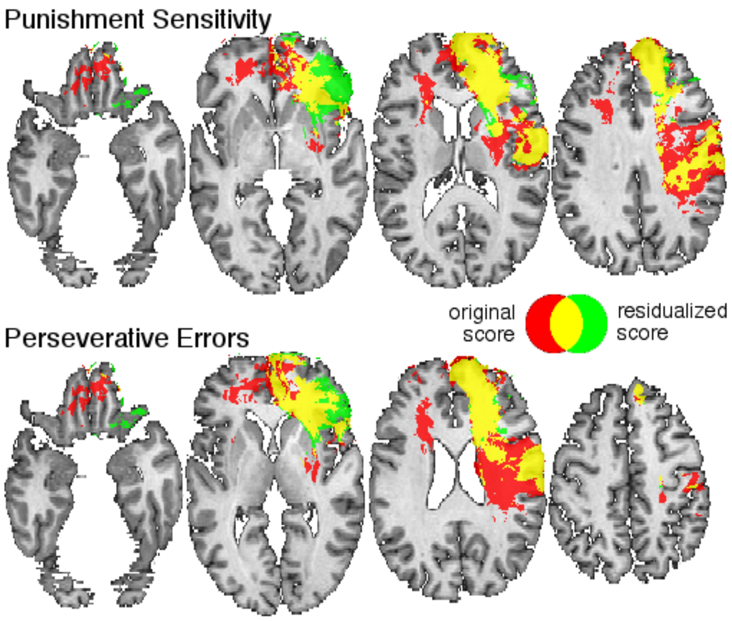

Supplementary Figure 6. VLSM analysis of the residualized measures of punishment sensitivity (P) and perseverative errors (PSV) with demographic (gender, handedness, education), neuroanatomical (lesion volume) and neuropsychological background assessment (see tests in Table 1C) removed. The residuals for P reached significance at 1% FDR, whereas the residuals for PSV were significant only at the reduced threshold of 5% FDR.

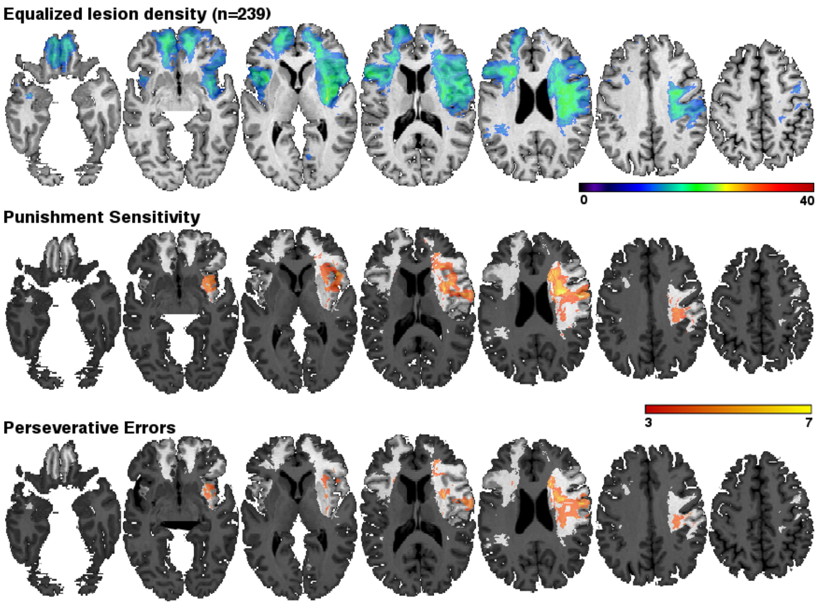

Supplementary Figure 7. VLSM analysis for a subsample of n=239 patients with equalized lesion distribution in bilateral PFC (~ 20 lesions in each ventral PFC voxel). Analysis was conducted for voxels with a minimum overlap of n=12 lesions. Voxels with lower Ns (<12) and less power to detect a lesion-deficit effect are shaded in gray. Punishment sensitivity and perseverative errors exhibit a similar right PFC involvement as in the original analysis with 328 patients, but no left PFC involvement despite an equalized lesion density in bilateral PFC.

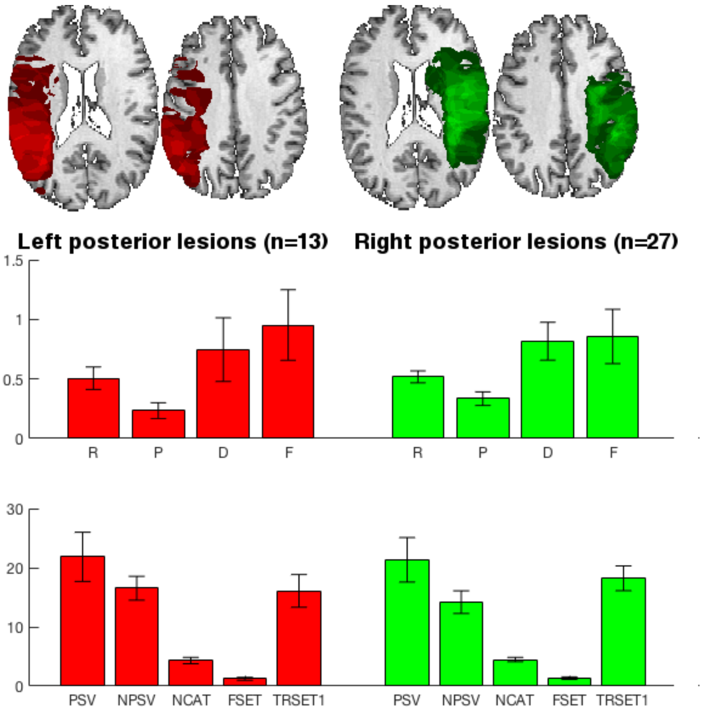

Supplementary Figure 8. Comparison of profiles of model parameters and WCST scores for 13 patients with left posterior lesions (some of whom had language impairments, but were able to give valid WCST performances) and 27 patients with right posterior lesions (error bars are ± 1s.e.m.).

Supplementary Note 6: Cluster analysis

To identify subgroups that exhibited distinct profiles of model parameters we conducted a 2-stage k-means cluster analysis and used the mean of the silhouette values as a criterion for selecting the number of clusters and appropriate distance measure. The silhouette value is a measure of distance of each point in a cluster to the points in the neighboring clusters. It ranges from +1 (very distant to points in neighboring cluster) over 0 (equidistant to neighboring clusters) to -1 (very close to points in neighboring cluster, probably a misclassification). The mean silhouette value succinctly summarizes the overall goodness of separation between clusters.

Table S1 shows the average silhouette value for different distance measures and varying numbers of clusters when using the model parameters of all 328 participants as input data. The best model (Euclidean distance, k=3) is highlighted in red. Figure S8 shows the silhouette plot for this solution. There were no misclassifications in any cluster, but two clusters contained only 36 and 28 participants, respectively. The average model parameters and WCST scores in each of these clusters revealed quite different profiles and lesion locations (see Figure 5 in the main text), ranging from essentially non-impaired participants (judging from PSV and NPSV errors) with lesions in left and right anterior temporal lobe (cluster 1, n=36), to a large group of participants with both left and right prefrontal cortex and posterior lesions with a noticeable degree of impairment (cluster 2, n=266), to severely impaired participants with lesions in the right prefrontal cortex as well as right posterior regions (cluster 3, n=26). Essentially, this first k-means cluster analysis differentiated 3 groups by their broadly different degrees of impairment, but produced no further differentiation within the prefrontal cortex. In addition, because of the small sample sizes in clusters 1 and 3, it is difficult to draw definitive conclusions for these groups.

Supplementary Table 3. Average silhouette values for different k-mean clustering solutions (k=2-7) and different distance measures (correlation, cityblock, Euclidean). The largest average silhouette value is shown in red and was selected for analysis.

distance measures	k = 2	k = 3	k = 4	k = 5	k = 6	k = 7
**Correlation**	0.6090	0.7221	0.6509	0.6385	0.6100	0.6300
**Cityblock**	0.3569	0.3118	0.3748	0.3450	0.3207	0.2960
**Euclidean**	0.7530	**0.8080**	0.5901	0.5223	0.5159	0.5013

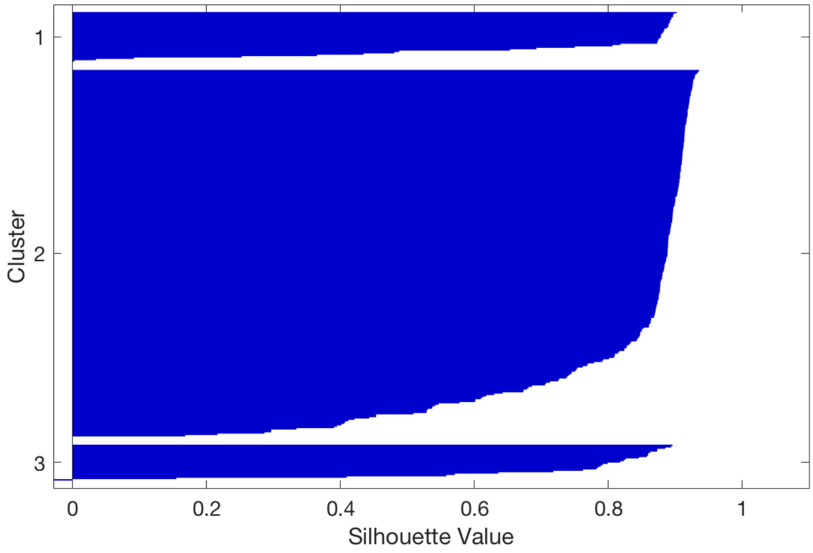

Supplementary Figure 9. Silhouette Plot for the best clustering solution (Euclidean distance, k=3) in the first cluster analysis. Highest silhouette values were above 0.8, indicating good separation between clusters; there were no misclassifications.

We then submitted the data of cluster 2 to another k-means cluster analysis to determine if we could identify further subgroups within the group of patients with PFC lesions who may have been lumped together in the first cluster analysis. Table S2 shows the average silhouette values for this second cluster analysis and Figure S9 shows the silhouette plot for this solution. We detected again 3 clusters with substantial sample sizes (cluster 2a: n=95, cluster 3b: n=93, cluster 2c: n=76). Average model parameters and WCST scores for these clusters are shown in Figure 6 in the main text. Although the density maps of all clusters revealed the involvement of right prefrontal cortex, we found that the extent of peak lesion density (n ≥ 10) in the PFC was different between the clusters 2a-c: 2a showed the smallest extent of right PFC involvement, 2b showed the largest, whereas 2c was in the middle. This order was also associated with impairments on the WCST (as measured with PSV and NPSV errors): participants in cluster 2b had the largest number of PSV errors, followed by participants in cluster 2c; participants in cluster 2a showed the fewest PSV errors.

Supplementary Table 4. Average silhouette values for different k-means clustering solutions (k=2-7) and different distance measures (correlation, cityblock, Euclidean) in the second cluster analysis on the data from cluster 2. The largest average silhouette value is show in red and was selected for analysis.

distance measures	k = 2	k = 3	k = 4	k = 5	k = 6	k = 7
**Correlation**	0.5862	**0.6676**	0.6074	0.5558	0.4937	0.5357
**Cityblock**	0.3476	0.3143	0.3000	0.2668	0.2679	0.2705
**Euclidean**	0.5244	0.5016	0.4470	0.4387	0.4022	0.4214

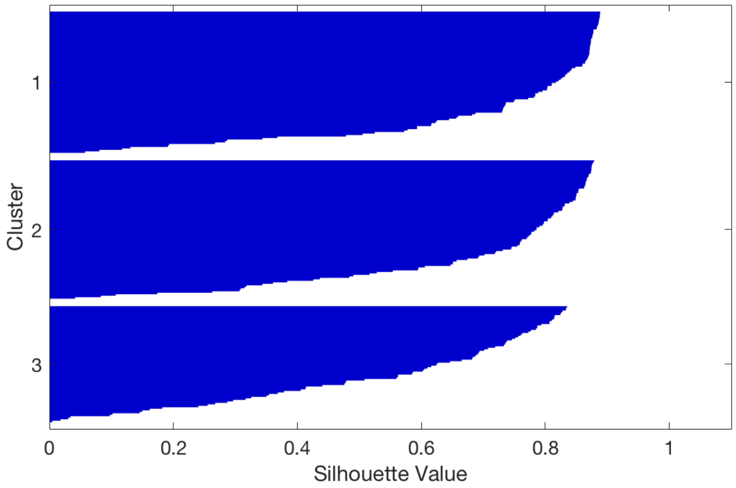

Supplementary Figure 10. Silhouette Plot for the best clustering solution (Euclidean distance, k=3) in the second cluster analysis. Highest silhouette values were above 0.8, indicating good separation between clusters; there were no misclassifications.

Supplementary Note 7: Cross-validation analysis of clustering solution
We checked the reliability and validity of our clustering solution by conducting a cross-validation analysis, drawing randomly 1000 samples of 50% of our participants and applying the same 2-step cluster analysis (3-means cluster algorithm). The sizes of the clusters are remarkably similar to those from the full sample (divided by 2) and the overlap of the cross-validation density maps is generally moderate to high.

Supplementary Table 5. Cross-validation to verify stability of clustering solution.

Cluster No.		Full sample (divided by 2)	Cross-validation samples	Percent overlap of cross-validation density map
**1**		36 (18)	19.3	73.3 %
**2**		266 (133)	131.4	87.3 %
	**2a**	95 (47.5)	37.4	65.8 %
	**2b**	93 (46.5)	52.6	47.4 %
	**2c**	78 (39)	41.4	57.9 %
**3**		26 (13)	13.5	6.2 %

Next, we compared the average profile of model parameters and WCST scores of the 1000 cross-validation samples (represented as bars in Figure S11) to those from the full sample (represented as horizontal blue lines). This demonstrates that the original profile of model parameters and WCST scores replicates to a very high degree across the cross-validation samples, suggesting our clustering analysis yielded replicable and valid solutions.

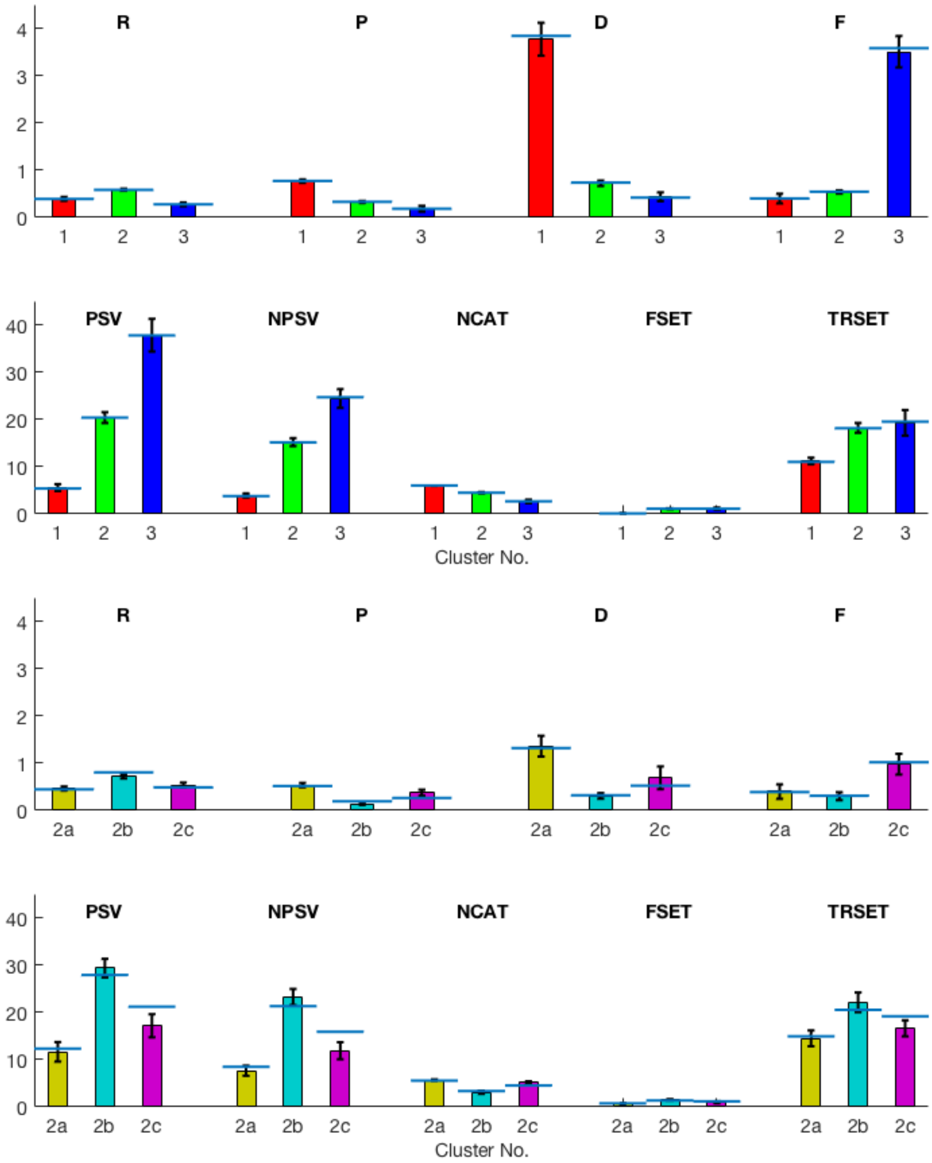

Supplementary Figure 11. Model parameters and WCST scores of 1000 cross-validation samples comprising 50% of the participants from the original sample, drawn randomly with replacement. Each cross-validation sample was submitted to the same 2 clustering analyses as in the original analysis of the full sample. Mean data from the cross-validation samples are shown as bars. Horizontal lines represent the data from the clustering analysis of the full sample descried in the main text (error bars are ± 1s.e.m.).

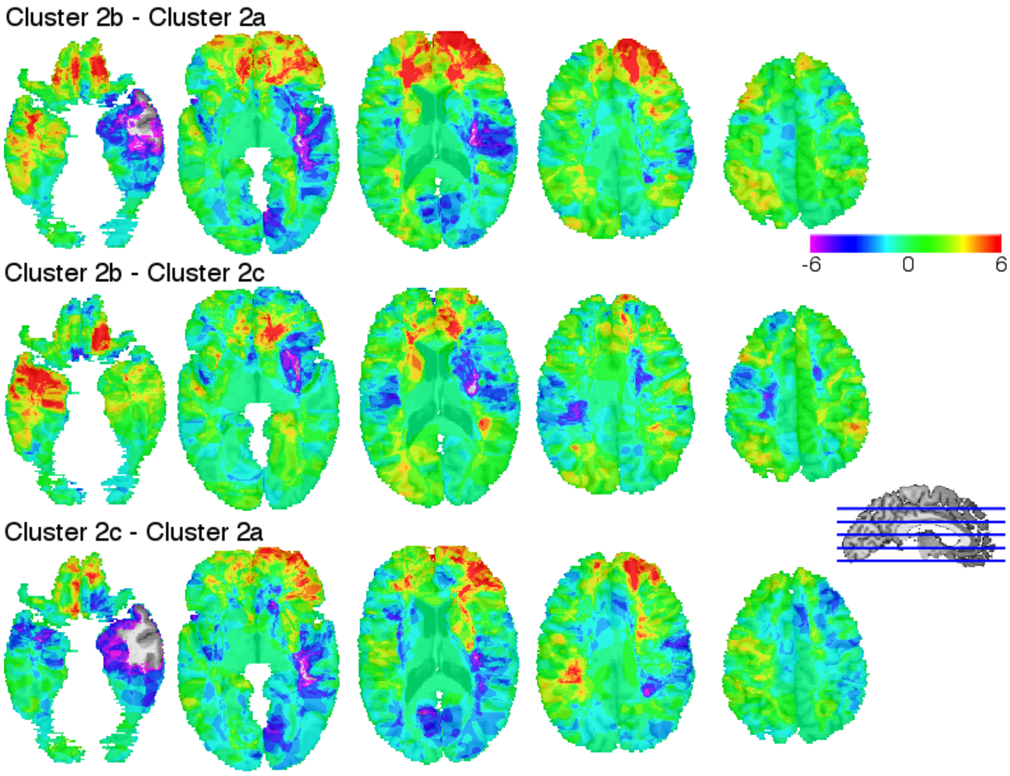

Supplementary Figure 12. Pairwise difference density maps for clusters 2a-c. Red areas indicate positive differences between the lesion density maps of the clusters (i.e., greater lesion density in the first of the 2 clusters). Blue regions reveal negative differences (i.e., greater lesion density in the second of the 2 clusters).

References

1. Heaton, R. K., Chelune, G. J., Talley, J. L., Kay, G. G. & Curtis, G. *Wisonsin Card Sorting Test (WCST). Manual revised and expanded*. (Psychological Assessment Resources, 1993).
2. Frank, R. J., Damasio, H. & Grabowski, T. J. Brainvox: An interactive, multimodal visualization and analysis system for neuroanatomical imaging. *Neuroimage* **5,** 13–30 (1997).
3. Fiez, J. A., Damasio, H. & Grabowski, T. J. Lesion segmentation and manual warping to a reference brain: Intra - and interobserver reliability. *Hum Brain Mapp* **9,** 192–211 (2000).
4. Bishara, A. J. *et al.* Sequential learning models for the Wisconsin card sort task: Assessing processes in substance dependent individuals. *Journal of Mathematical Psychology* **54,** 5–13 (2010).
5. Shiffrin, R. M., Lee, M. D., Kim, W. & Wagenmakers, E.-J. A survey of model evaluation approaches with a tutorial on hierarchical bayesian methods. *Cogn Sci* **32,** 1248–1284 (2008).
6. Plummer, M. JAGS: A program for analysis of Bayesian graphical models using Gibbs sampling. in (2003).
7. Lee, M. D. & Wagenmakers, E. J. *Bayesian Cognitive Modeling: A Practical Course*. 1–274 (Cambridge University Press, 2014).
8. Bates, E. *et al.* Voxel-based lesion-symptom mapping. *Nature Neuroscience* **6,** 448–450 (2003).
9. Brunner, E. & Munzel, U. The Nonparametric Behrens - Fisher Problem: Asymptotic Theory and a Small - Sample Approximation. *Biometrical Journal* **42,** 17–25 (2000).
10. Nichols, T. & Hayasaka, S. Controlling the familywise error rate in functional neuroimaging: a comparative review. *Stat Methods Med Res* **12,** 419–446 (2003).
11. Rorden, C., Karnath, H.-O. & Bonilha, L. Improving lesion-symptom mapping. *J Cogn Neurosci* **19,** 1081–1088 (2007).
12. Medina, J., Kimberg, D. Y., Chatterjee, A. & Coslett, H. B. Inappropriate usage of the Brunner-Munzel test in recent voxel-based lesion-symptom mapping studies. *Neuropsychologia* **48,** 341–343 (2010).
